# Healthcare Workers Attitudes, Practices and Sources of Information for COVID-19 Vaccination: An Italian National Survey

**DOI:** 10.3390/ijerph19020733

**Published:** 2022-01-10

**Authors:** Francesca Papini, Sara Mazzilli, Dania Paganini, Lucia Rago, Guglielmo Arzilli, Angelo Pan, Antonio Goglio, Benedetta Tuvo, Gaetano Privitera, Beatrice Casini

**Affiliations:** 1 Department of Translational Research and New Technologies in Medicine and Surgery, University of Pisa, 56127 Pisa, Italy; f.papini1@studenti.unipi.it (F.P.); sara.mazzill@gmail.com (S.M.); d.paganini1@studenti.unipi.it (D.P.); lucia.r.med@gmail.com (L.R.); guglielmo.arzilli@gmail.com (G.A.); b.tuvo@studenti.unipi.it (B.T.); gaetano.privitera@med.unipi.it (G.P.); 2 Scuola Normale Superiore, 56126 Pisa, Italy; 3 Infectious Diseases ASST Cremona, 26100 Cremona, Italy; a.pan@asst-cremona.it; 4 Scientific Board of the Italian Multidisciplinary Society for the Prevention of Infections in Healthcare Organizations (SIMPIOS), 20159 Milano, Italy; agoglio44@gmail.com

**Keywords:** COVID-19 vaccination, SARS-CoV-2, healthcare workers, vaccine hesitancy, immunization campaign, sources of information

## Abstract

**Background:** Vaccination of healthcare workers (HCWs) is a crucial element to overcome the COVID-19 pandemic. The aim of this survey was to assess attitudes, sources of information and practices among Italian Healthcare workers (HCWs) in relation to COVID-19 vaccination. **Methods:** From 19 February to 23 April 2021, an anonymous voluntary questionnaire was sent to the mailing list of the main National Health Service structures. Data were collected through the SurveyMonkey platform. **Results:** A total of 2137 HCWs answered. Hesitancy towards COVID-19 vaccination was more frequent in females, in those with lower concern about COVID-19, and in nurses, auxiliary nurses (AN) and healthcare assistants. Hesitant professionals were more likely to not recommend vaccination to their patients or relatives, while a high concern about COVID-19 was related to an increased rate of recommendation to family members. HCWs were mostly in favor of mandatory vaccination (61.22%). Female sex, a lower education level, greater hesitancy and refusal to adhere to flu vaccination campaigns were predictors influencing the aversion to mandatory vaccination. All categories of HCWs referred mainly to institutional sources of information, while scientific literature was more used by professionals working in the northern regions of Italy and in infection control, infectious diseases, emergencies and critical areas. HCWs working in south-central regions, nurses, AN, healthcare technicians, administrators and HCWs with a lower education level were more likely to rely on internet, television, newspapers, and the opinions of family and friends. **Conclusions:** Communication in support of COVID-19 immunization campaigns should consider the differences between the various HCWs professional categories in order to efficiently reach all professionals, including the most hesitant ones.

## 1. Introduction

In March 2020, the World Health Organization (WHO) declared the COVID-19 pandemic. Despite the efforts made to face the emergency, the virus has taken a dramatic toll in terms of human lives and economic burden [1].

Since the beginning of the pandemic, many efforts have been made by the scientific community to counteract the advance of SARS-CoV-2. Despite the many therapies tested, prevention through vaccination has been the key to changing the course of the epidemic. In June 2021, the European Medicines Agency approved four vaccines: Comirnaty, COVID-19 Vaccine Janssen, Spikevax and Vaxzevria. Vaccination campaigns started in many European countries prioritizing high risk groups, including healthcare workers (HCWs).

HCWs are a multifaceted workforce defined by WHO as “all people engaged in actions whose primary intent is to enhance health”, including doctors, nurses, auxiliary nurses, social and health workers, paramedical staff, laboratory and radiology technicians and all supporting professional figures involved in assistance activities, such as hospital administrators and community workers [2]. Being at the forefront of emergency response, HCWs are inevitably exposed to a higher risk of contracting the infection, risking their health and becoming carriers towards patients and others [3].

As of 27 August 2021, in Italy, 37,046,307 people were vaccinated, of whom 1,852,026 were HCWs [3]. Considering the present evidence, the COVID-19 vaccination campaign has shown evidence of significantly reducing the incidence of cases, hospitalizations and deaths [4,5,6].

The Italian National Institute of Health (ISS) reported that 136,872 HCWs had tested positive for severe acute respiratory syndrome coronavirus 2 (SARS-CoV-2) in Italy. The infected HCWs accounted for 3.12% of the total number of positive cases, as of 1 September 2021 [7].

Taking this prevalence into account, it is clear that the protection of HCWs is crucial to ensure the essential levels of assistance needed during incoming pandemic waves in order to provide direct patient care and support to operational services [7]. Furthermore, HCWs play a key role in vaccine promotion and patient guidance: the literature reports a direct link between the favorable vaccine attitude of HCWs and the vaccination coverage of their patients [8]. For that reason, the spread of concerns about vaccine efficacy and safety among HCWs can hinder the success of the vaccination campaign [9,10].

According to the weekly report of Italian Ministry of Health, as 27 August 2021, 35,039 HCWs (1.79%) were not vaccinated against COVID-19 [11]. Since 1 April 2020, the Italian Government has mandated COVID-19 vaccination for all HCWs [12].

Several studies have analyzed the factors associated with hesitancy towards COVID-19 vaccination in HCWs [13,14].

Vaccine hesitancy is a behavior that includes the refusal of vaccines or the delaying of vaccination despite available services. The WHO identified HCWs as a high-priority group for COVID-19 vaccination. The WHO regarded vaccine hesitancy as a global health threat in 2019. Vaccine hesitancy in the public has also been linked to the level of vaccine hesitancy among HCWs [15,16].

However, to the best of our knowledge, few studies were focused on sources of information used by HCWs to acquire knowledge on the COVID-19 vaccine.

As reported in the literature, the variables that can cause greater hesitation towards COVID-19 vaccination by healthcare professionals are those relating to the demographic characteristics of the population under study (age, sex, educational qualification and area of residence) as well as the knowledge, perception and attitudes towards the COVID-19 vaccine and the behaviors they have towards the flu vaccination [17,18,19,20].

This study, promoted by the Italian Multidisciplinary Society for the Prevention of Infections in Healthcare Organizations (SIMPIOS), explores Italian HCWs’ attitudes (hesitancy towards COVID-19 vaccination and attitudes towards mandatory vaccination), practices (advice to patients and family members) and sources of information concerning COVID-19 vaccination in order to identify predictors of hesitancy that can be addressed for the success of the vaccination campaign among this heterogeneous population.

## 2. Materials and Methods

### 2.1. Study Setting

This study was based on a national cross-sectional survey conducted between the 19 February 2021 and the 23 April 2021. An e-mail invitation containing the link to the anonymous and voluntary questionnaire was sent through the mailing list of the national scientific society SIMPIOS to hospital health departments, directorates of the main health structures of the National Health Service and Local Health Units asking to forward it to the HCWs. The questionnaire was hosted electronically on SurveyMonkey (SurveyMonkey Inc., San Mateo, CA, USA).

### 2.2. Questionnaire Development

The survey developed by the research team consisted of five parts: personal information (age, gender, geographical area of service, length of service, job), concern about COVID-19 disease, acceptance of influenza vaccination during the 2018/2019, 2019/2020 and 2020/2021 campaigns, attitude and practices toward COVID-19 vaccination and sources of information used by HCWs to acquire knowledge on the COVID-19 vaccine.

The questionnaire was composed of 24 questions and included categorical responses, open-ended questions and 5-point Likert scales [21] (2 levels of agreement, 1 neutral choice, 2 levels of disagreement) (Supplementary Materials). To assess the adequacy of the tool, especially in terms of the questions’ comprehensibility, a pilot study was conducted, and the questionnaire was revised according to the remarks of the participating HCWs.

A dichotomous variable about hesitancy was created starting from the question: “If you think you are not vaccinating yourself against COVID-19, can you indicate the reasons among those listed?”. Respondents who claimed they would refuse COVID-19 vaccine for health-related reasons (clinical contraindication, advice from a physician or having already contracted COVID-19) or who claimed they would accept the vaccine if they had had the opportunity were classified as “non-hesitant”. Respondents were classified as “hesitant” if they selected one of the following answers: “Because the COVID-19 vaccine had too short a period of testing and control”, “I am afraid of side effects”, “I prefer to wait until more people have been vaccinated, or to wait until next year”, “I doubt it is effective”, “COVID-19 is not a serious disease/the symptoms are generally mild at least in my age group”, “It is preferable to develop physiological immunity (after exposure to the disease)” or “Serious side effects due to the COVID-19 vaccine are kept under wraps” (Supplementary Materials, Questionnaire). Furthermore, a participant was considered vaccine hesitant if he or she answered No or Don’t Know to one of the following proposals: “Would you recommend the COVID-19 vaccine to your patients?” or “Would you recommend the COVID-19 vaccine to your family members?”.

If a participant answered, ‘yes’ to one of these proposals, he/she was not considered to be ‘vaccine hesitant’.

### 2.3. Statistical Analysis

The software Stata (version 13.0) was used for the statistical analyses. We started with a descriptive analysis of the main characteristics of the sample. The second level of analysis was completed in three stages. We analyzed the following dependent variables: (i) acceptance of flu vaccine in 2020/2021; (ii) hesitancy toward the COVID-19 vaccine; (iii) being against mandatory COVID-19 vaccination; (iv) recommending COVID-19 vaccine to patients; (v) recommending COVID-19 vaccine to relatives; and (vi) using different sources of information. First, comparisons between proportions of each independent variable category by dependent variable were carried out using the Pearson Chi-square test or the Fisher exact test, in case any expected frequency was lower than five. Then, univariate logistic regression analysis was carried out to explore the association between each independent variable and the different outcomes of interest. All independent variables found to be associated at a *p*-value less than 0.05 during the univariate analyses were entered in the multivariate logistic regression. Finally, multivariate logistic regression models were constructed to identify factors significantly associated with the dependent variables. To build multivariate models, a manual stepwise variables’ selection procedure was used in order to assess confounding and effect modification. To select the variables included in the models, we ran the Likelihood-ratio test. All reported values are two-sided, and a value of *p* ≤ 0.05 was used as a threshold for statistical significance for all analyses.

## 3. Results

### 3.1. Participant Characteristics

A total of 2137 Italian health professionals responded to the questionnaire. The principal characteristics of the study group are shown in Table 1.

The response rate to the questionnaire was not the same in all regions. However, the geographical distribution (northern–central–southern) of respondents followed the geographical distribution of HCWs employed in Italy.

### 3.2. Concern about COVID-19

Most of the respondents showed a medium to high level of concern about COVID-19 (2010/2124, 94.63%), while only 5.37% (114/2124) had a low or very low level of concern.

### 3.3. HCWs Adherence to Influenza Vaccination (in 2018, 2019 and 2020 Seasons)

Starting from the 2018–2019 campaign, an increasing influenza vaccination rate among respondents was registered. The increase was minimal in the 2019–2020 campaign (∆% = 13.16), while it was significant in the 2020–2021 campaign (∆% = 71.44).

**Figure 1:**
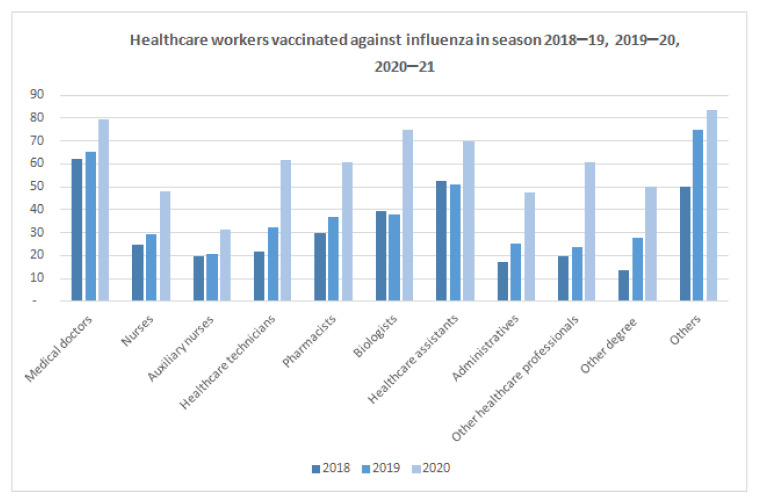
HCWs vaccinated against flu in 2018–2019, 2019–2020 and 2020–2021 seasons.

The largest increases were recorded among professionals with non-medical education degrees (∆% = 81.25) and other health professions (∆% = 154.55), as well as among administrative staff (∆% = 87.87), biologists (∆% = 97.37), pharmacists (∆% = 65.22) and healthcare technicians (∆% = 90.97) (Figure 1).

According to the multivariate logistic regression results, male gender was correlated with lower likelihood of refusing the influenza vaccination in the 2020–21 season (OR = 0.63; CI 95% 0.50–0.81; *p* < 0.01). HCWs working in hospital surgical areas (OR = 1.40; CI 95% 1.05–1.85; *p* = 0.02) had a higher likelihood of refusing the vaccination in the 2020–21 season compared to the ones working in hospital medical areas. Nurses (OR = 3.22; CI 95% 2.46–4.21; *p* < 0.01), other healthcare professionals (OR = 2.40; CI 95% 1.31–4.26; *p* < 0.01), auxiliary nurses, (OR = 4.73; CI 95% 2.79–8.02; *p* < 0.01), healthcare technicians (OR = 2.44; CI 95% 1.57–3.78; *p* < 0.01) and those without healthcare-related degrees (OR = 4.08; CI 95% 1.26–13.23; *p* = 0.02) had a higher likelihood of refusing the vaccination in the 2020–21 season when compared with medical doctors. HCWs with more than 35 years of service (OR = 0.44; CI 95% 0.29–0.69; *p* < 0.01) and HCWs working in central regions (OR = 0.60; CI 95% 0.47–0.77; *p* < 0.01) appeared to be more likely to be vaccinated.

### 3.4. COVID-19 Vaccine Hesitancy

Overall, 6.76% (144/2131) of the participants were hesitant about COVID-19 vaccines. According to the results of the multivariate logistic regression model, males were less hesitant than females (OR 0.37; CI 95% 0.14–0.98; *p* = 0.04). Respondents with a high and very high level of concern about COVID-19 disease had a lower likelihood of being hesitant. Nurses, auxiliary nurses and healthcare assistants showed a higher likelihood of being hesitant (Table A1).

### 3.5. Attitudes towards Mandatory COVID-19 Vaccination

Overall, 62.69% (1304/2080) of our respondents were in favor of mandatory vaccination against COVID-19.

According to the results of the multivariate logistic regression model, male gender was associated with a lower likelihood of being against mandatory vaccination, as well as respondents with a higher level of education. Conversely, hesitant respondents and respondents who had not been vaccinated for influenza in the 2020–2021 season had a higher likelihood of being against mandatory vaccination (more details in Table A1).

### 3.6. Recommendation of COVID-19 Vaccination to Patients

A very low percentage of respondents would not have recommended the COVID-19 vaccination to their patients (0.88%; 18/2052). According to the results of the multivariate logistic regression model, hesitant responders, auxiliary nurses and healthcare assistants had a higher likelihood of not recommending COVID-19 vaccination to their patients. (Table A2).

### 3.7. Recommendation of COVID-19 Vaccination to Family Members

A low percentage of respondents would not have recommended the COVID-19 vaccination to relatives (1.66%; 35/2113). According to the results of the multivariate logistic regression model, hesitant responders had a higher likelihood of not recommending vaccination to their relatives. Respondents with a medium, high or very high level of concern about COVID-19 disease were more likely to recommend vaccination to their relatives (Table A2).

### 3.8. Sources of Information

**Figure 2:**
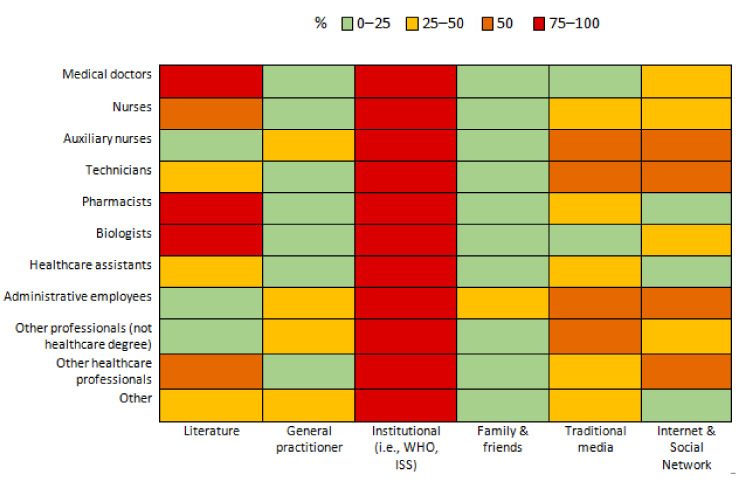
Percentages of health professionals who obtained information through different sources.

The institutional sources of information (i.e., the WHO and the ISS) were the most used by HCWs to gather information on COVID-19. Figure 2 shows the percentage of health professionals who searched for information through different sources.

Nurses and auxiliary nurses had a higher likelihood of using digital media (web sites and social media), traditional media (e.g., television and newspapers), advice from family and friends, and institutional sources to obtain information on COVID-19. They had a lower likelihood of using scientific literature.

Technicians and administrative employees had a higher likelihood of using traditional media, advice from family and friends, and institutional sources, while they had a lower likelihood of using scientific literature. However, administrative employees had a higher likelihood of using digital media, while technicians had a lower likelihood of using that source of information. HCWs employed in emergency and critical areas or in infection control and infectious disease departments had a higher likelihood of obtaining information from scientific literature compared with those working in chirurgical areas of hospitals. On the contrary, they had a lower likelihood of trusting digital media, traditional media, and advice from family and friends. Respondents from southern regions and islands had a lower likelihood of using institutional sources of information and scientific literature. In contrast, they had a higher likelihood of using advice from family and friends, digital media and traditional media as sources of information.

Respondents with a higher education had a higher likelihood of using institutional sources of information and a lower likelihood of using traditional media. Younger HCWs (Age ≤ 35) had a higher likelihood of using digital media and advice from family and friends. Male HCWs had a higher likelihood of using digital media and traditional media (Table A3).

## 4. Discussion

The aim of this study was to evaluate, through a national cross-sectional survey, attitudes, practices and sources of information among Italian HCWs in relation to COVID-19 vaccination.

The WHO defined vaccine hesitancy as a refusal or delay in vaccine acceptance: it represents a major public health challenge, affecting even those who work in the healthcare sector [21,22].

In our study, 6.76% of respondents were found to be hesitant to the COVID-19 vaccine, in line with what was previously reported in the literature for Italian HCWs [13]. Worldwide, the prevalence of COVID-19 vaccination hesitancy in HCWs is not uniformly diffused, varying from 4.3 to 72% [23].

Gender, occupation and education were found to be significantly associated with COVID-19 vaccine hesitancy. In particular, females were more hesitant than males and this was confirmed by other studies on HCWs and in the general population [24,25]. This may be due to the fact that men have an increased risk perception and a higher acceptance of pharmaceutical measures [24]. These data are of particular relevance given the prevalence of female workers in the Italian healthcare workforce [26].

Among the different healthcare professionals who adhered to the survey, nurses, healthcare assistants and auxiliary nurses showed greater vaccine hesitancy compared to physicians. These findings may be due to the heterogeneity of backgrounds, personal attitudes and expertise of HCWs, and highlight a need for tailored communication strategies [27].

Respondents from the same professional categories that were associated with hesitance about the COVID-19 vaccine also had a higher likelihood of having refused the influenza vaccination in the 2020–21 season.

Respondents who had a greater vaccine acceptance were more likely to be concerned about COVID-19: our data are in keeping with data found in the literature for health care professionals and the general population [24,28]. Notably, our findings are confirmed by a recent study on vaccine hesitancy in United Kingdom HCWs, which found common predictors of vaccine hesitancy to be female sex, non-medical occupation, lack of influenza vaccination and lower perceived risk of COVID-19: the mentioned study focuses on ethnicity and religious beliefs that have less relevance in the Italian HCWs, who are more homogeneous in terms of these parameters [29].

Italy was the first European nation to introduce mandatory COVID-19 vaccination. At the time the survey was started, COVID-19 vaccination was not already mandatory. Nevertheless, the majority of the respondents (62.69%) agreed on mandating COVID-19 vaccination and this is similar to what has been reported for influenza vaccination. Hesitant HCWs were more likely to disagree on mandatory vaccination [30].

Education also seemed to play a role in determining acceptance of mandatory vaccination: HCWs with a master’s degree or a postgraduate specialist were in favor of mandatory vaccination.

Adhesion to previous flu vaccine campaigns was associated with a higher likelihood of accepting the COVID-19 vaccine and being in favor of mandatory vaccination.

According to our data, HCWs’ adherence to the influenza vaccination campaign in the 2020–21 season increased significantly compared to previous years, reaching 69.86% of the total. This trend follows what had already emerged from studies in the general population and HCWs [31]. This may be due to several reasons, although the COVID-19 pandemic scenario may have increased adherence to the flu vaccination campaign [32].

We found that only a small percentage of respondents (1.66%) would not recommend the COVID-19 vaccine to their relatives, and even fewer respondents (0.88%) would not recommend it to their patients. This is in contrast with the overall ratio of hesitant healthcare workers (6.76%), which is higher; thus, there is a dichotomy between the courses of action healthcare providers would choose for themselves and for their patients. Furthermore, the only factor impacting both kinds of recommendation was hesitancy, which coheres with the findings of other studies, but surprisingly, only the advice for relatives was affected by the HCWs’ concern about COVID-19 [9,33]. This result may be due to the fact that the emotional dimension is a relevant issue in HCW’s decision-making process.

Regarding sources of information used, national and international institutions were the most trusted sources of information across all professional categories. In particular, institutional sources of information were more used by those with a higher level of educational attainment; this could also be due to the fact that people with a higher level of education generally have greater health literacy [3].

People with lower health literacy have greater difficulty in evaluating and differentiating low quality health information from high quality health information [34,35,36]. During the COVID-19 pandemic, the spread of misinformation (false and misleading information) was facilitated by a rise in the use of online discussion forums and social media platforms, which allowed it to travel faster and wider than ever before. As reported by “Countering online vaccine misinformation in the EU/EEA”, misinformation about COVID-19 was greater than other diseases due to the much higher number of posts (68% of all posts identified) [37].

Data from our study show that traditional media, web sites and social media, and recommendations from friends or family were less used as sources of information by those with a higher education. They were more used by nurses, auxiliary nurses, and technicians with a health role.

Scientific literature seemed to be disproportionately consulted by biologists, doctors, pharmacists and nurses, in line with the data that emerged from the literature, as all these professionals require continuous updating on epidemiology and disease management guidelines [38].

Scientific literature was also used more by professionals who work in areas most affected by the pandemic, such as infection control and hospital hygiene, infectious diseases, and emergency and critical areas.

Our study has several limitations:

- The number of participants is limited. Although online surveys are powerful tools to obtain responses from a great mass of people in a short space of time, during the pandemic, it was difficult to involve healthcare professionals in this survey due to their extensive involvement in the care of COVID-19 patients. In administering the questionnaire, the possibility of duplicate responses was excluded. For this reason, the data were not entirely representative but could nevertheless be indicative of attitudes and practices of HCWs regarding COVID-19 vaccination.
- The presented data comprised theoretical information about attitudes to vaccines given that at the time the survey was started, COVID-19 vaccination was not yet mandatory. We therefore wanted to assess what the tendency towards vaccination was. It is essential to regularly monitor the attitudes and practices of healthcare professionals toward COVID-19 vaccines not only because of their role in vaccination campaigns, but also, of course, because they are involved in patient care.
- Regarding the source of the information, the recommendations of family and friends or acquaintances category did not consider their level of education.
- As the study was not causal but descriptive, we did not group the survey’s answers into hesitancy categories, and we did not apply any of the known conceptual models for grouping vaccine hesitancy determinants, such as the “3 Cs” model, which highlights the three categories (complacency, convenience and confidence) [14].

## 5. Conclusions

HCWs constitute a heterogeneous group of professionals with different backgrounds and experience that are indispensable in the fight against COVID-19.

Our study shows that several variables could predict HCWs’ attitudes towards COVID-19 vaccination, the practices regarding patients and their choices of reliable sources of information.

It is worth noting the preponderance of HCWs recommending COVID-19 vaccination to patients and family members and showing a positive attitude in favor of mandatory vaccination, consistent with regulatory provisions in Italy.

One of the key elements in the successful introduction of a vaccine is the high-quality training of all HCWs about the new vaccine and the disease it prevents [39]. Moreover, communication is particularly necessary to achieve high vaccination coverage in hard-to-reach populations, such as potentially hesitant individuals [39].

Communication in support of immunization campaigns should consider the differences among professional categories in order to effectively reach the most hesitant professionals. It is also fundamental to make good use of mass media with correct and effective messages.

## Figures and Tables

**Table 1:** Characteristics of the survey respondents.

	N	%	South and Islands	499	23.35
**Gender**	2131		**Education**	2126	
Male	603	28.30	Secondary school	60	2.82
Female	1528	71.70	High school	389	18.30
**Age range**	2131		Bachelor’s degree	548	25.78
<31	190	8.92	Master’s degree	322	15.15
31–40	440	20.65	Post-graduate education	807	37.96
41–50	571	26.79	**Profession**	2120	
51–60	700	32.85	Medical doctors	634	29.91
>60	230	10.79	Nurses	894	42.17
**Geographic area**	2130		Auxiliary nurses	100	4.72
North	933	43.66	Technicians	189	8.92
Centre	698	32.66	Pharmacists	24	1.13
Biologists	53	2.50		N	%
Healthcare assistants	41	1.93	Territorial medicine	74	3.50
Administrative employees	85	4.01	Administration	111	5.26
Other healthcare professionals	62	2.92	Other	64	3.03
Other	38	1.80	**Length of service**	2121	
**Working area**	2112		<6	420	19.80
Hospital chirurgical areas	382	18.09	6–10	166	7.83
Hospital medical areas	637	30.16	11–15	275	12.97
Infection control	111	5.26	16–20	226	10.66
Diagnostic and services	372	17.61	21–25	278	13.11
Health management	117	5.54	26–30	256	12.07
Emergency and critical areas	140	6.63	31–35	273	12.87
Infectious disease departments	104	4.92	>35	227	10.70

## Data Availability

All data is contained in the manuscript.

## Supplementary Materials

The following supporting information can be downloaded at: https://www.mdpi.com/article/10.3390/ijerph19020733/s1.

## Appendix A

**Table A1:** Multivariate logistic regression models for COVID-19 vaccine hesitancy (0: non-hesitant; 1: hesitant) and for opinion on mandatory COVID-19 vaccination (0: in favor; 1: against).

	Hesitancy				Mandatory COVID-19 Vaccine			
	OR	Conf. Int. 95%		*p*-Value	OR	Conf. Int. 95%		*p*-Value
**Gender**								
Male	1.00				1.00			
Female	0.37	0.14	0.98	0.04	0.75	0.59	0.94	0.02
**Education**								
Secondary school					1.00			
High school					0.94	0.47	1.87	0.86
Bachelor’s degree					0.79	0.40	1.54	0.49
Master’s degree					0.49	0.24	0.98	0.04
Post-graduate education					0.49	0.25	0.96	0.04
**Concern about COVID-19**								
Very low	1.00				1.00			
Low	0.71	0.11	4.58	0.72	2.05	0.70	6.02	0.19
Moderate	0.23	0.04	1.19	0.08	1.01	0.39	2.64	0.98
High	0.12	0.02	0.64	0.01	0.81	0.31	2.12	0.67
Very High	0.17	0.03	1.02	0.05	0.56	0.21	1.52	0.26
**Hesitancy**								
Not hesitant					1			
Hesitant					5.20	3.09	8.77	0.00
**Profession**								
Medical doctors	1.00							
Nurses	2.62	1.10	6.22	0.03				
Auxiliary nurses	10.05	3.38	29.91	0.00				
Technicians	1.48	0.30	7.31	0.63				
Pharmacists								
Biologists	2.04	0.24	17.37	0.51				
Healthcare assistants	10.42	2.42	44.95	0.00				
Administrative employees	2.06	0.23	17.37	0.52				
Other professionals (not holding healthcare degrees)								
Other healthcare professionals	3.65	0.72	18.50	0.12				
Other								
**Flu vaccine in 2020/2021**								
Yes					1			
No					1.95	1.58	2.41	0.00

**Table A2:** Multivariate logistic regression models for recommendation of COVID-19 vaccine to patients (0: yes; 1: no) and for recommendation of COVID-19 vaccine to relatives (0: yes; 1: no).

	Recommendation of COVID-19 Vaccine to Patients				Recommendation of COVID-19 Vaccine to Relatives			
	OR	Conf. Int. 95%		*p*-Value	OR	Conf. Int. 95%		*p*-Value
**Concern about COVID-19**								
Very low					1.00			
Low					0.78	0.14	4.33	0.78
Moderate					0.18	0.04	0.79	0.02
High					0.07	0.01	0.36	0.00
Very High					0.10	0.01	0.74	0.02
**Hesitancy**								
Not hesitant	1				1			
Hesitant	12.90	7.26	69.23	0.00	76.77	31.70	185.88	0
**Profession**								
Medical doctors	1.00							
Nurses	2.72	0.31	23.58	0.36				
Auxiliary nurses	13.28	1.37	128.59	0.03				
Healthcare assistants	21.28	1.69	268.13	0.02				
Other healthcare professionals	7.06	0.41	122.88	0.18				

**Table A3:** Multivariate logistic regression models for the use source of information (0: no; 1: yes).

	Literature N = 1972				Internet N = 1819				Traditional Media N = 1976				Family & Friends N = 1810				Institutional (i.e., WHO, ISS) N = 1978			
	OR	Conf. Int. 95%		*p*-Value	OR	Conf. Int. 95%		*p*-Value	OR	Conf. Int. 95%		*p*-Value	OR	Conf. Int. 95%		*p*-Value	OR	Conf. Int. 95%		*p*-Value
**Geographic area**																				
North	1.00				1.00				1.00				1.00				1.00			
Centre	0.65	0.51	0.83	0.00	1.44	1.14	1.81	0.00	1.38	1.10	1.74	0.01	1.47	0.96	2.26	0.08	0.90	0.67	1.20	0.46
South and Islands	0.56	0.43	0.74	0.00	1.35	1.04	1.74	0.02	1.56	1.21	2.01	0.00	2.14	1.34	3.49	0.00	0.53	0.40	0.72	0.00
**Working area**																				
Hospital chirurgical areas	1.00				1.00				1.00				1.00				1.00			
Hospital medical areas	0.98	0.73	1.30	0.87	1.09	0.83	1.44	0.52	1.07	0.81	1.39	0.64	1.10	0.70	1.73	0.67	0.82	0.59	1.12	0.21
Infection control	2.42	1.38	4.24	0.00	0.68	0.42	1.08	0.10	0.37	0.22	0.63	0.00	0.35	0.10	1.17	0.09	8.47	2.61	27.51	0.00
Diagnostic and services	0.87	0.62	1.23	0.44	0.80	0.58	1.11	0.19	0.87	0.63	1.19	0.37	0.62	0.33	1.16	0.13	1.11	0.76	1.63	0.58
Health management	0.80	0.50	1.30	0.37	0.54	0.33	0.88	0.01	0.72	0.46	1.14	0.16	0.69	0.26	1.82	0.45	2.49	1.28	4.84	0.01
Emergency and critical areas	1.55	1.03	2.33	0.04	0.55	0.37	0.84	0.01	0.37	0.24	0.57	0.00	0.39	0.17	0.90	0.03	1.65	0.96	2.84	0.07
Infectious disease departments	2.32	1.30	4.16	0.01	0.40	0.24	0.65	0.00	0.35	0.20	0.61	0.00	0.25	0.07	0.85	0.03	2.20	1.17	4.11	0.01
Territorial medicine	0.65	0.37	1.14	0.13	0.86	0.49	1.52	0.61	1.12	0.66	1.90	0.68	0.44	0.12	1.58	0.21	1.56	0.78	3.11	0.21
Administration	1.04	0.57	1.87	0.91	1.03	0.56	1.88	0.93	0.69	0.39	1.23	0.21	0.68	0.22	2.13	0.51	1.49	0.69	3.18	
**Age class**																				
<31	1.00				1.00								1.00							
31–40	0.72	0.48	1.07	0.10	0.58	0.40	0.85	0.01					0.34	0.20	0.58	0.00				
41–50	0.68	0.47	1.01	0.06	0.57	0.39	0.83	0.00					0.43	0.26	0.70	0.00				
51–60	1.07	0.61	1.74	0.75	0.31	0.21	0.45	0.00					0.15	0.08	0.26	0.00				
>60	1.03	0.61	1.74	0.91	0.37	0.23	0.60	0.00					0.16	0.06	0.42					
**Gender**																				
Female					1.00				1.00											
Male					1.26	1.00	1.59	0.05	1.35	1.07	1.69	0.01								
**Profession**																				
Medical doctors	1.00				1.00				1.00				1.00				1.00			
Nurses	0.29	0.20	0.40	0.00	1.50	1.17	1.94	0.00	1.77	1.29	2.44	0.00	1.85	1.10	3.11	0.02	2.03	1.34	3.07	0.00
Auxiliary nurses	0.14	0.07	0.26	0.00	1.79	1.10	2.91	0.02	1.68	0.94	2.98	0.08	7.76	3.74	16.12	0.00	2.92	1.41	6.04	0.00
Technicians	0.29	0.18	0.46	0.00	0.53	1.29	3.00	0.00	2.84	1.83	4.40	0.00	3.81	1.78	8.14	0.00	1.50	0.85	2.62	0.16
Biologists	1.31	0.52	3.27	0.57	1.03	0.53	2.02	0.92	1.15	0.56	2.35	0.70	2.08	0.55	7.90	0.28	5.91	1.38	25.31	0.02
Pharmacists	2.27	0.51	10.0	0.28	0.58	0.20	1.65	0.30	1.30	0.51	3.29	0.59	3.05	0.64	14.64	0.16	6.86	0.90	52.23	0.06
Healthcare assistants	0.22	0.10	0.48	0.00	0.57	0.25	1.32	0.19	0.86	0.39	1.92	0.73	4.10	1.24	13.55	0.02	2.55	0.88	7.41	0.09
Administrative employees	0.35	0.13	0.95	0.04	5.67	1.99	16.13	0.00	2.59	0.97	6.94	0.06	8.16	1.94	34.37	0.00	1.35	0.43	4.23	0.61
Other healthcare professionals	0.26	0.14	0.47	0.00	1.61	0.91	2.87	0.10	1.78	0.99	3.22	0.06	1.45	0.46	4.58	0.53	1.98	0.87	4.50	0.10
Other professionals (not holding healthcare degrees)	0.19	0.06	0.45	0.00	1.83	0.55	6.05	0.32	4.15	1.43	11.99	0.01	3.21	0.35	29.13	0.30	1.33	0.27	6.52	0.72
Other	0.18	0.03	1.09	0.06					0.30	0.03	2.76	0.29					1.22	0.20	7.57	0.83
**Education**																				
Secondary school									1.00								1.00			
High school									0.86	0.45	1.61	0.63					1.37	0.66	2.83	0.39
Bachelor’s degree									0.62	0.32	1.20	0.16					2.41	1.13	5.12	0.02
Master’s degree									0.49	0.25	0.99	0.05					2.99	1.32	6.77	0.01
