# Supplementary material for: Healthcare Workers Attitudes, Practices and Sources of Information for COVID-19 Vaccination: An Italian National Survey
Source: Int J Environ Res Public Health. 2022 Jan 10;19(2):733. doi: 10.3390/ijerph19020733 (PMC8775438; doi:10.3390/ijerph19020733)
Supplement: Supplementary file 1 [file ijerph-19-00733-s001.zip › ijerph-1503567-supplementary.pdf]

Supplementary Materials

S1: Questionnaire

Survey Covid-19 across Italian Healthcare Workers

1. **SEX:**
   - ☐ Male
   - ☐ Female

2. **AGE :**
   - ☐ < 31
   - ☐ 31-40
   - ☐ 41-50
   - ☐ 51-60
   - ☐ 60

3. **LENGHT OF SERVICE (YEARS):**
   - ☐ < 6
   - ☐ 6-10
   - ☐ 11-15
   - ☐ 16-20
   - ☐ 21-25
   - ☐ 26-30
   - ☐ 31-35
   - ☐ > 35

4. **EDUCATION:**
   - ☐ Elementary school
   - ☐ Secondary school leaving certificate
   - ☐ High school diploma
   - ☐ Bachelor degree
   - ☐ Master degree
   - ☐ Post-graduate diploma or a specializing master
   - ☐ Other (specify)

5. **PROFESSION**
   - ☐ Medical doctor
   - ☐ Nurse
   - ☐ Healthcare assistant
   - ☐ Midwife
   - ☐ Pharmacist
   - ☐ Biologist
   - ☐ Psicologist
   - ☐ Other degree (physicists, chemists, engineers, ...)
   - ☐ Healthcare technician
   - ☐ Auxiliary nurse (Healthcare operator)
   - ☐ Other (specify)

6. AREA OF SERVICE
- ☐ Health management
- ☐ Hospital hygiene and infection control
- ☐ Occupational medicine
- ☐ Infectious diseases
- ☐ Medical area
- ☐ Surgical area
- ☐ Nursing home
- ☐ Territorial medicine
- ☐ Administration
- ☐ Other (specify)

7. PLACE OF WORK (CITY)

8. PLACE OF WORK (REGION)

9. IN THE PAST YEARS HAVE YOU SUBJECTED TO ANTI-FLU VACCINATION?

	Yes	No
2018 -2019	☐	☐
2019 – 2020	☐	☐
2020 – 2021	☐	☐

10. HAVE YOU BEEN IN CONTACT WITH SUSPECTED OR CERTIFIED CASES COVID-19?
- ☐ Yes
- ☐ No

11. HAVE YOU WORKED / DO YOU WORK IN A COVID DEPARTMENT?
- ☐ Yes
- ☐ No

12. AMONG THOSE INDICATED BELOW, DO YOU THINK YOU HAVE RISK FACTORS FOR SERIOUS MANIFESTATIONS DUE TO COVID-19? MULTIPLE ANSWERS ARE POSSIBLE
- ☐ None
- ☐ Diabetes
- ☐ Obesity
- ☐ Cardiovascular diseases
- ☐ Autoimmune diseases
- ☐ Lung diseases

13. HOW DO YOU ASSESS THE FOLLOWING STATEMENTS?

True False Don’t Know

- Obesity is a risk factor for the severe form of COVID-19 at all ages: ☐ ☐ ☐
- Belonging to the age group ≥ 50 - 60 years is the main risk factor to develop a severe form of covid-19 ☐ ☐ ☐
- Prolonged symptoms (fatigue, anxiety ...) are often reported as consequences of COVID-19 infection ☐ ☐ ☐

14. **HAVE YOU EVER BEEN INFECTED BY COVID-19?**
    - ☐ No
    - ☐ Yes, asymptomatic, treated at home
    - ☐ Yes, symptomatic, hospitalized
    - ☐ Yes, serious, admitted to intensive care

15. **WHAT IS YOUR LEVEL OF CONCERN REGARDING COVID-19?**
    - ☐ Very low
    - ☐ Low
    - ☐ Medium
    - ☐ High
    - ☐ Very high

16. **HOW DO YOU ASSESS THE LEVEL OF YOUR KNOWLEDGE ABOUT COVID-19 AND VACCINES?**

	Very low	Low	Medium	High	Very high
• about COVID-19:	☐	☐	☐	☐	☐
• about vaccination:	☐	☐	☐	☐	☐
• about COVID-19 vaccination:	☐	☐	☐	☐	☐

17. **INDICATE WHICH OF THE FOLLOWING SOURCES OF INFORMATION ABOUT VACCINATION FOR COVID-19 WERE MOST IMPORTANT FOR YOU (NO MORE THAN FIVE OF THE BELOW)**
    - ☐ Family
    - ☐ Friends or acquaintances' recommendation
    - ☐ TV
    - ☐ Internet
    - ☐ Social network
    - ☐ Newspapers (printed or on line)
    - ☐ Scientific literature
    - ☐ General practicioner
    - ☐ International institutions (WHO or others)
    - ☐ National institutions (Ministery of Heatlh, Istituto Superiore di Sanità)
    - ☐ Health service

18. **ASSESS THE INFORMATION QUALITY ABOUT COVID-19 VACCINATION IN RELATION TO THE SOURCES OF INFORMATION LISTED ABOVE**

	Very low	Low	Medium	High	Don't know
Family:	☐	☐	☐	☐	☐
Friends or acquaintances' recommendation :	☐	☐	☐	☐	☐
TV:	☐	☐	☐	☐	☐
Internet:	☐	☐	☐	☐	☐
Social network	☐	☐	☐	☐	☐
Newspapers (printed or on line) :	☐	☐	☐	☐	☐
Scientific literature:	☐	☐	☐	☐	☐
General practicioner:	☐	☐	☐	☐	☐
International institutions (WHO or others) :	☐	☐	☐	☐	☐
National institutions (Ministery of health, ISS) :	☐	☐	☐	☐	☐
Health service:	☐	☐	☐	☐	☐

19. **IF YOU THINK YOU ARE NOT VACCINATING YOURSELF AGAINST COVID-19, CAN YOU INDICATE THE REASONS AMONG THOSE LISTED? (MULTIPLE ANSWERS POSSIBLE)**
- ☐ Because the COVID-19 vaccine had too short a period of testing and control
- ☐ I'm afraid of side effects
- ☐ I prefer to wait until more people have been vaccinated, or to wait until next year
- ☐ I doubt it's effective
- ☐ Covid-19 is not a serious disease / the symptoms are generally mild at least in my age group
- ☐ It is preferable to develop physiological immunity (after exposure to the disease)
- ☐ Clinical contraindication
- ☐ I have already had the disease
- ☐ Advice from a physician
- ☐ Serious side effects due to the COVID-19 vaccine are kept under wraps

20. **WOULD YOU RECOMMEND THE COVID-19 VACCINE TO YOUR PATIENTS?**
- ☐ Yes
- ☐ No
- ☐ Don't Know

21. **WOULD YOU RECOMMEND THE COVID-19 VACCINE TO YOUR FAMILY MEMBERS?**
- ☐ Yes
- ☐ No
- ☐ Don't Know

22. **DIFFERENT VACCINES ARE AVAILABLE OR NEAR AVAILABLE. CHOOSE AN ANSWER:**
- ☐ One vaccine is as good as another, having been approved by regulatory agencies (EMA, AIFA)
- ☐ It is preferable that the person receiving the vaccine can choose which vaccine to be injected
- ☐ The person receiving the vaccine must be able to choose which vaccine to be injected
- ☐ The choice of the type of vaccine must be made by the Health Authorities

23. WHAT DO YOU THINK ABOUT THE MANDATORY COVID-19 VACCINATION FOR HEALTHCARE WORKERS?

- ☐ It must be mandatory
- ☐ Can be recommended, but cannot be mandatory
- ☐ Other (specify)

24. OBSERVATIONS, CRITICS, PROPOSALS?
